# Synergistic Effects of Licorice Root and Walnut Leaf Extracts on Gastrointestinal Candidiasis, Inflammation and Gut Microbiota Composition in Mice

**DOI:** 10.1128/spectrum.02355-21

**Published:** 2022-03-09

**Authors:** Hélène Authier, Valérie Bardot, Lucile Berthomier, Bénédicte Bertrand, Claude Blondeau, Sophie Holowacz, Agnès Coste

**Affiliations:** a Geroscience and Rejuvenation Research Center (RESTORE), UMR 1301-Inserm 5070-CNRS EFS, Université P. Sabatier, Toulouse, France; b PiLeJe Industrie, Saint-Bonnet-de-Rochefort, France; c PiLeJe Laboratoire, Paris, France; University of Debrecen

**Keywords:** candidiasis, gut inflammation, licorice, plant extract, prebiotic, walnut, microbiota

## Abstract

Candida albicans is an opportunistic pathogen that causes gastrointestinal (GI) candidiasis closely associated with intestinal inflammation and dysbiosis. Drug resistance, side effects of available antifungal agents, and the high recurrence of candidiasis highlight the need for new treatments. We investigated the effects of hydroethanolic extracts of licorice root (LRE) and walnut leaf (WLE) on GI colonization by C. albicans, colon inflammation, and gut microbiota composition in C57BL/6 female mice. Oral administration of LRE and WLE alone or in combination once daily for 12 days before C. albicans infection and then for 5 days after infection significantly reduced the level of C. albicans in the feces of gastrointestinal infected mice as well as colonization of the GI tract, both extracts showing robust antifungal activity. Although total bacterial content was unaffected by the extracts (individually or combined), the abundance of protective bacteria, such as *Bifidobacterium* spp. and Faecalibacterium prausnitzii, increased with the combination, in contrast to that of certain pathobiont bacteria, which decreased. Interestingly, the combination induced a more robust decrease in the expression of proinflammatory genes than either extract alone. The anti-inflammatory activity of the combination was further supported by the reciprocal increase in the expression of anti-inflammatory cytokines and the significant decrease in enzymes involved in the synthesis of proinflammatory eicosanoids and oxidative stress. These findings suggest that LRE and WLE have synergistic effects and that the LRE/WLE combination could be a good candidate for limiting GI candidiasis and associated inflammation, likely by modulating the composition of the gut microbiota.

**IMPORTANCE** The adverse effects and emergence of resistance of currently available antifungals and the high recurrence of candidiasis prompt the need for alternative and complementary strategies. We demonstrated that oral administration of hydroethanolic extracts of licorice root (LRE) and walnut leaf (WLE) separately or in combination significantly reduced the colonization of the gastrointestinal (GI) tract by C. albicans, highlighting a robust antifungal activity of these plant extracts. Interestingly, our data indicate a correlation between LRE and WLE consumption, in particular the combination, and a shift within the gut microbiome toward a protective profile, a decrease in colonic inflammation and prooxidant enzymes, suggesting a synergistic effect. This study highlights the significant prebiotic potential of the LRE/WLE combination and suggests that the health benefits are due, at least in part, to their ability to modulate the gut microbiota, reduce inflammation and oxidative stress, and protect against opportunistic infection.

## INTRODUCTION

Candida albicans inhabit the gastrointestinal (GI) tract of most healthy individuals (1, 2). As a commensal member of the microbiota, the yeast is generally harmless, but it can become an opportunistic pathogen, particularly in individuals with impaired immunity (1). C. albicans is a major cause of infections worldwide; it commonly triggers superficial mucosal infections and may also, under favorable conditions, lead to potentially life-threatening deep tissue infections (1).

The GI tract is a key reservoir of C. albicans, and the fungus is well adapted to growth in the environment provided by the GI tract and to the changes that can take place within this, for example, following the use of antibiotics (2, 3). In addition, C. albicans has been associated with several GI diseases, such as celiac disease and inflammatory bowel diseases (3–7). C. albicans is thought to exacerbate inflammatory processes due to a sequence of mutually perpetuating events, including dysbiosis and low-grade inflammation in the gut that sustains the growth of the fungus while its excessive growth fosters further inflammation, increasing lesions and delaying healing (2, 3, 6). C. albicans is, therefore, considered to be involved in the pathogenesis of certain gastrointestinal diseases.

The emergence of drug resistance, the adverse effects of available antifungal agents, and the high recurrence rate of candidiasis have necessitated a search for new therapies. This has led to an increased interest in exploring the potential of plants for the treatment of fungal infections (8, 9).

The antifungal potential of licorice (Glycyrrhiza glabra L.) and walnut (Juglans regia L.) among other plants have been evaluated in several *in vitro* and *in vivo* studies. A propylene glycol extract of a dry powder of licorice roots inhibited the growth of C. albicans *in vitro* (10). Other studies investigated major phytochemical compounds extracted from licorice roots, such as the saponin glycyrrhizin, the aglycone of glycyrrhizin 18β-glycyrrhetinic acid, the chalcone licochalcone A, and the isoflavonoid glabridin (11–17). All these studies have contributed to demonstrating the antifungal potential of these compounds and thus of licorice root against C. albicans infections. For example, in an *in vivo* study with glycyrrhizin, mice were inoculated with C. albicans at lethal doses with or without previous administration of glycyrrhizin at the dose of 0.5 mg/kg/day for 15 to 20 days. Prior administration of glycyrrhizin decreased the mortality rate from 100% to 65%. Mean survival time increased from 7 to 11 days and symptom severity decreased (11).

There are fewer studies on the antifungal effects of a walnut leaf. The antifungal activity of walnut has been reported in a few *in vitro* studies evaluating different types of extracts. A hydromethanolic extract of walnut leaf was found to be the most effective of the plant extracts tested *in vitro* against C. albicans and other *Candida* species (18), confirming previous observations in studies investigating methanol, ethyl acetate, diluted acetone (19), and hydroethanolic extracts (20). It should be noted that in a study assessing aqueous extracts of different walnut leaf cultivars, no effect was observed on the tested fungi (C. albicans and C. neoformans) and Gram-negative bacteria species (Escherichia coli, Pseudomonas aeruginosa, and Klebsiella pneumoniae). Only the growth of Gram-positive bacteria (Bacillus cereus, B. subtilis, and Staphylococcus aureus) was inhibited by these extracts (21).

In addition to their direct effects on C. albicans and their immunomodulatory properties, plants and their secondary metabolites have been shown to have prebiotic effects (22–27). These could be of interest in the context of GI candidiasis and other GI diseases given the demonstrated links between these diseases, *Candida*, inflammation, and dysbiosis. Among plant-derived compounds, phenolics, which encompass structural variants of flavonoids, hydroxybenzoic acids, hydroxycinnamic acids, coumarins, stilbenes, ellagitannins, and lignans can modify the composition of the gut microbiota (23, 24). The prebiotic potential of licorice root extracts has been evidenced *in vitro* (24, 25). Recent findings suggest that licorice (Glycyrrhiza uralensis Fisch.) could correct overall gut microbial dysbiosis and fecal metabolic disorders associated with CPT-11-induced colitis in mice (26). Compounds known to be present in walnut leaves, e.g., hydroxycinnamic acids and flavonoids, have also been reported to modulate gut microbiota composition (23, 24, 27).

Although many studies showed the *in vitro* antifungal effect against *Candida* sp. of licorice root extract and compounds (10, 14–16), only a few *in vivo* studies demonstrate its effect on candidiasis (11, 12, 17). Concerning walnut leaf, its antimicrobial effect was only demonstrated in *in vitro* assays (18–21). The objective of this study was to evaluate *in vivo* antifungal effects of specific hydroethanolic extracts of a walnut leaf (walnut leaf extract [WLE]) and licorice root (licorice root extract [LRE]), both separately and particularly in combination, in mice with GI candidiasis with the intention also to investigate whether the observed effects could involve anti-inflammatory activity and modulation of gut microbiota.

## RESULTS

### Phytochemical analysis of LRE revealed the presence of glycyrrhizin and several other compounds.

**FIG 1:**
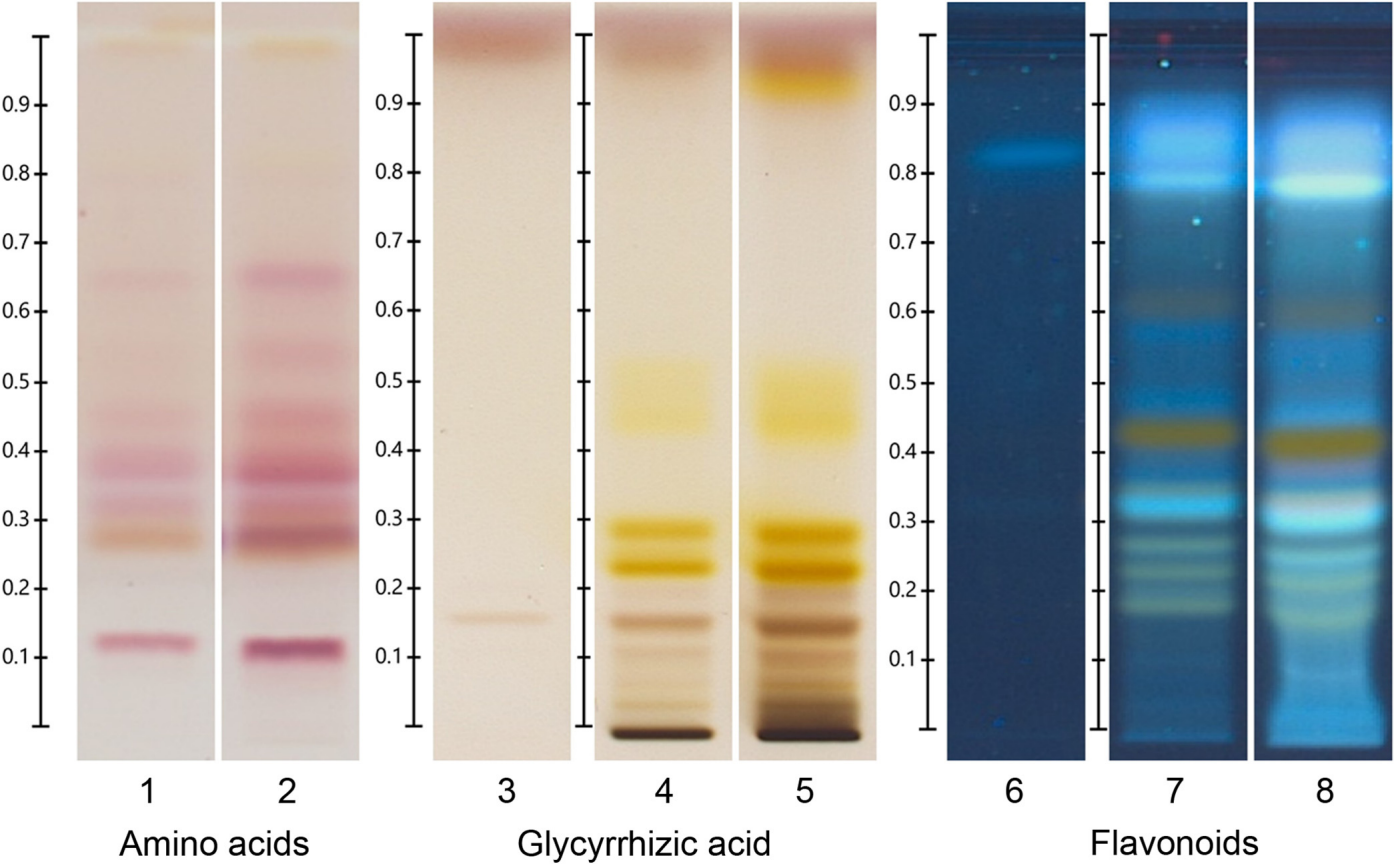
High-performance thin-layer chromatography (HPTLC) analysis. Track 1: Extract of ground roots of Glycyrrhiza glabra (2 μL); track 2: licorice root extract (LRE) (2 μL); track 3: Glycyrrhizic acid (2 μL); track 4: Extract of ground roots of G. glabra (2.5 μL); track 5: LRE (2.5 μL); track 6: Formononetine (5 μL); track 7: Extract of ground roots of G. glabra (2 μL); track 8: LRE (2 μL).

**FIG 2:**
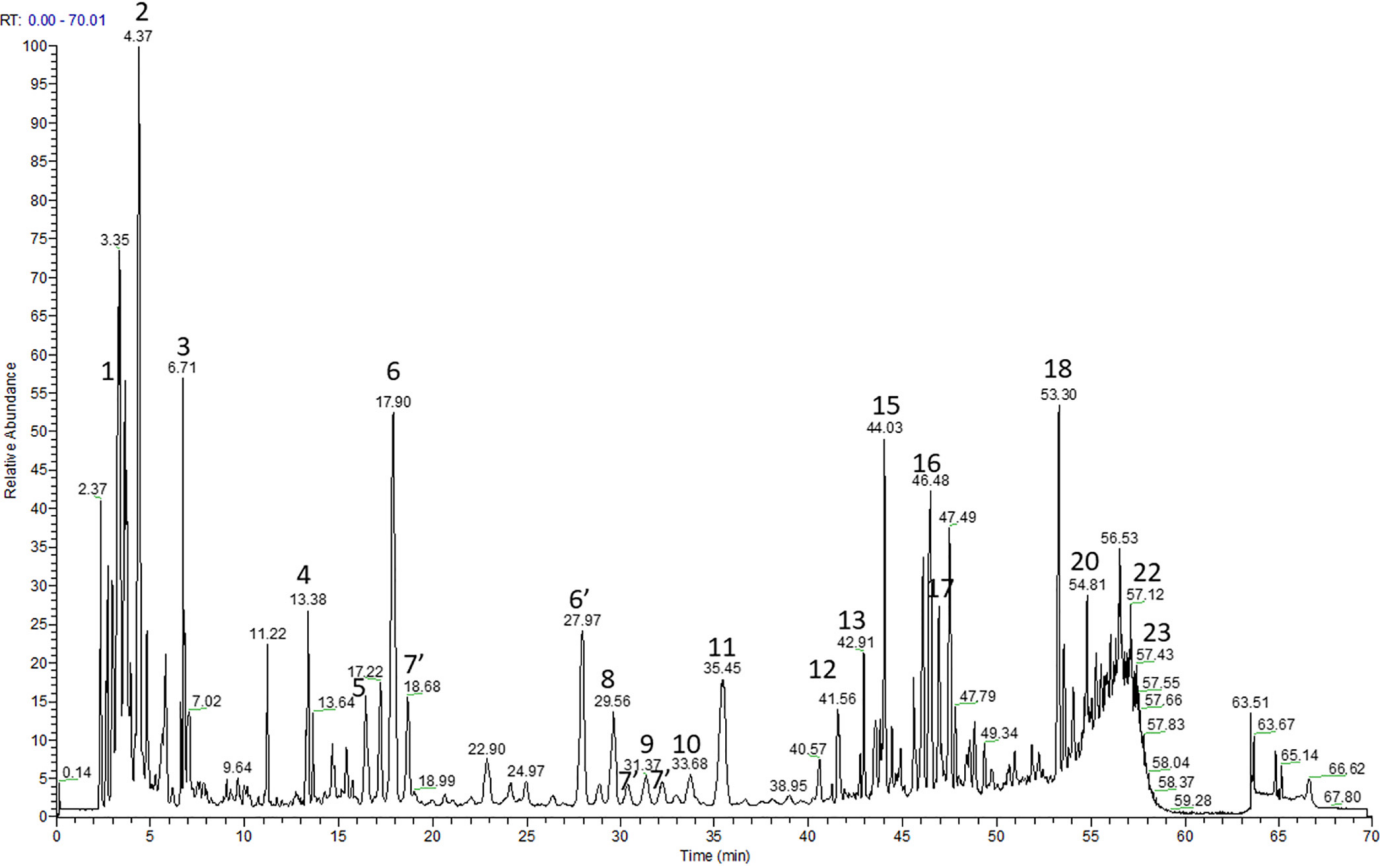
Liquid chromatography-mass spectrometry (LC-MS) analysis in negative ionization mode.

High-performance thin-layer chromatography (HPTLC) analyses identified both glycyrrhizic acid (glycyrrhizin) and formononetin in LRE (Fig. 1). Ultra-high-performance liquid-chromatography–tandem mass spectrometry (UHPLC-MS) analysis (Fig. 2 and Table 1) confirmed the presence of glycyrrhizic acid (Fig. 2, peak 13) in LRE and identified enoxolone (glycyrrhetinic acid; peak 23). Various other acids, including citric acid (peak 3) and p-hydroxy benzyl malonic acid (peak 4) were also identified as well as flavonoids, such as liquiritin apioside and isoliquiritin apioside (peaks 6 and 6’), licuroside (peak 8), isoviolanthin (peak 5), 3-hydroxyglabrol (peak 21), and glabrol (peak 22).

**TABLE 1:** Compounds identified by liquid chromatography-mass spectrometry (LC-MS) in negative ionization mode

No.	t_R_	Compound	Formula	Mass	Ion m/z M-H theoretical	M-H (MS)	M-H (MS/MS)	Reference	M-H standard or ref (MS/MS)
1	3.2	Glucose	C_6_H_12_O_6_	180.06339	179.0561	179.0555	(179)59/71/89/113/101/85	(51)	(179)59/89/71/119/101/113/85
2	4.37	Sucrose	C_12_H_22_O_11_	324.11621	341.1089	341.1088	(341)89/59/71/119/179/113/ 101/161/143/131	(52)	(341)71/89/101/179/59/113/119/161/85/143/131
3	6.71	Citric acid	C_6_H_8_O_7_	192.0270	191.0197	191.0186	(191)111/87/85/129/57/113/173	Standard	(191)111/87/85
4	13.38	HBMA (p-hydroxybenzylmalonic acid)	C_10_H_10_O_5_	210.0528	209.0455	209.0450	(209)165/121/59/121/93	(53)	165/121
5	16.42	Isoviolanthin	C_27_H_30_O_14_	578.16356	577.1563	577.1566	(577)383/353/457/297/413	(54, 55) for fragmentation	457/503/473/559/383
6 and 6’	17.90 and 27.97	Liquiritin apioside or Isoliquiritin apioside	C_26_H_30_O_13_	550.16864	549.1614	549.1617 549.1617	(549)255/135/119/ 429/153/417/297	(53, 56) for fragmentation	255/429/297/417
7, 7’, 7’’, 7’’’	18.1, 18.6, 30.4, 32.21	Neoliquiritin or Liquiritin or Isoliquiritin	C_21_H_22_O_9_	418.12638	417.1191	417.1196 417.1193	(417)255/135/119/153/148	(53, 57) for fragmentation	135/119/255
8	29.56	Licuroside	C_26_H_30_O_13_	550.16864	549.1614	549.1618	(549)255/135/119/153/417/297	(53)	255/429/297/417
9	31.37	4′,7-dihydroxyflavone	C_15_H_10_O_4_	254.05791	253.0506	253.0504	(253)117/135/133/153/91/209	(58)	252/135/117
10	33.68	Licochalcone B	C_16_H_14_O_5_	286.08412	285.0768	285.0770	Positive (287)121/245/193/ 107/147/139	(59)	positive 255/193/165/121/93
11 and 11’	35.45 and 46.06	Liquiritigenin or Isoliquiritigenin	C_15_H_12_O_4_	256.07356	255.0663	255.0661	(255)119/135/153/91	(53, 60)	(255)213/161/153/135/91
12	41.56	Licoricesaponin J2	C_42_H_64_O_16_	824.41944	823.4122	823.4131	(823)351/113/193/85/71/ 72/75/59/175/99/289/ 647/761/235/333/ 307/471/805/261	(54)	805/779/761/647/539/351/333/289
13	42.91	Licoricesaponin A3	C_48_H_72_O_21_	984.45661	983.4493	983.4503	(983)351/113/821/193/85/71/72/75/59/99/645/803/289/627/235/759/469	(54)	923/863/821/803/760/645/351/289
14	43.46	Naringenin	C_15_H_12_O_5_	272.06847	271.0612	271.0614	(271)151/119/107/ 177/93/83/65	Standard	(579)271/151/459/119/177/107/235/316
15	44.03	24-hydroxyglycyrrhizin	C_42_H_62_O_17_	838.3987	837.3914	837.3920 837.3918 837.3922 837.3922	(837)351/113/193/85/175/71/ 75/99/103/289/485/661	(61)	819/781/776/>775/704/661/ 644/485/351/333/289
16	46.48	Glycyrrhizin (glycyrrhizic acid)	C_42_H_62_O_16_	822.40379	821.3965	821.3973 821.3971	(821)351/113/193/85/175/71/72/75/59/99/103/	(53, 62) for fragmentation	(821)351 /113 /193
17	46.96	Formononetin	C_16_H_12_O_4_	268.07356	267.0663	267.0664	(267)252/223/132/208/195	(54) Standard for fragmentation	(267)252/223/132/195
18	53.30	Glabridin	C_20_H_20_O_4_	324.13616	323.1289	323.1288	(323)/135/201/109/ 121/175/187/147/213	(53, 63) for fragmentation	135/201/21/121/147
19	53.38	Glabrone	C_20_H_16_O_5_	336.09977	335.0925	335.0925	(335)291/213/135/199	(54, 64) for fragmentation	291/320/213/292/307
20	54.81	Kanzonol Y	C_25_H_30_O_5_	410.20932	409.2020	409.2022 409.2020	(409)235/177/217/205/216/ 189/191/161/391	(54)	405/391/365/235/217
21	56.06	3-hydroxyglabrol	C_25_H_28_O_5_	408.19367	407.1864	407.1864	(407)235/177/216/205/161/389/233/229	(65)	201/185/177/161/349/215
22	57.12	Glabrol	C_25_H_28_O_4_	392.19876	391.1915	391.1919	(391)187/203/221/ 132/159	(66) for fragmentation	203/187/159
23	57.39	Enoxolone (glycyrrhetinic acid)	C_30_H_46_O_4_	470.33961	469.3323	469.3324	(469)425/355	(53, 67) for fragmentation	(469)425/355

### LRE and WLE effectively reduced fecal colonization and gastrointestinal C. albicans infection in mice.

**FIG 3:**
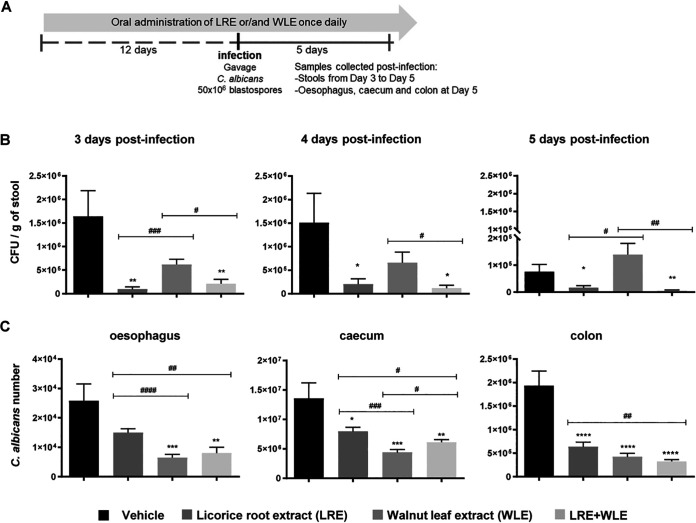
Effect of licorice root extract (LRE) and walnut leaf extract (WLE) alone or combined on the outcome of gastrointestinal candidiasis. (A) Experimental procedure. LRE and WLE were administered orally, separately (2.5 g/kg) or in combination (1.25 + 1.25 g/kg), once daily for 12 days before C. albicans infection and then for 5 days after infection. Esophageal and gastrointestinal candidiasis was established by gavage of C. albicans (*n* = 10 per group). Stools were collected daily from day 3 to 5 after infection to quantify viable C. albicans. After 5 days of infection, the mice were sacrificed and the esophagus, cecum, and colon were aseptically removed to evaluate C. albicans colonization, microbiota composition, and inflammatory status. (B) Numbers of viable C. albicans were determined by colonies forming unit (CFU) enumeration in stools collected 3, 4, and 5 days postinfection. (C) On day 5 postinfection, mice were sacrificed and C. albicans colonization in the esophagus, cecum, and colon were assayed by quantitative RT-PCR. Data are presented as means ± SEM. *, *P* ≤ 0.05; **, *P* ≤ 0.01; ***, *P* ≤ 0.005; ****, *P* ≤ 0.001 compared to Vehicle. ^#^, *P* ≤ 0.05; ^##^, *P* ≤ 0.01; ^###^, *P* ≤ 0.005; ^####^, *P* ≤ 0.001 compared between treatments.

To characterize the efficacy of plant extracts on the outcome of GI candidiasis, we evaluated the susceptibility of mice to *Candida* GI infection after oral administration of LRE and WLE separately or in combination (Fig. 3A).

We first evaluated the number of viable *Candida* in the stool that reflects both the colonization of the GI tract and the spontaneous yeast elimination following *Candida* oral administration. In accordance with a longer delay between *Candida* inoculation in mice and the day of the analysis of the fungal load in the stool, the number of viable yeast at day 5 compared to day 3 and day 4 postinfection was decreased (Fig. 3B).

When the two extracts were administered separately, although WLE tended to decrease the number of viable C. albicans in the feces from day 3 to day 4, only LRE achieved a significant decrease from day 3 to 5 postinfection. Interestingly, when the two plant extracts were administered together the number of viable C. albicans in the feces was substantially decreased from day 3 to 5 postinfection (Fig. 3B). The C. albicans loads in the esophagus, cecum, and colon at day 5 were significantly diminished in mice treated with LRE or WLE separately or in combination (Fig. 3C). Although WLE had no impact on the amount of *Candida* in the stool, the *Candida* colonization of the esophagus, cecum, and colon were significantly reduced at day 5 postinfection (Fig. 3C). Altogether, these results demonstrate that oral administration of LRE and/or WLE favors the clearance of C. albicans throughout the GI tract.

### Oral administration of LRE, WLE separately or in combination influenced the composition of the colonic mucosa-associated microbiota in mice subjected to GI candidiasis.

**FIG 4:**
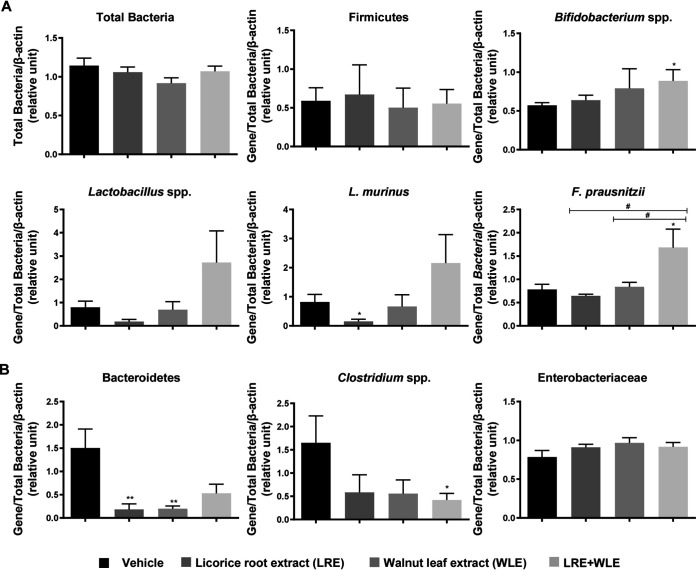
Effect of oral administration of licorice root extract (LRE) and walnut leaf extract (WLE) alone or in combination on the colon mucosa-associated microbiota of C. albicans-infected mice. The relative abundance of (A) protective and (B) pathobiont phyla and bacteria species in the colonic mucosa of C. albicans-infected mice treated with the LRE and WLE alone or in combination or with the vehicle (*n* = 10 mice per group) was evaluated by RT-PCR. Values were normalized to total bacteria and host β-actin. Data are presented as means ± SEM. *, *P* ≤ 0.05; **, *P* ≤ 0.01 compared to vehicle. ^#^, *P* ≤ 0.05 compared between treatments.

We evaluated the composition of colonic mucosa-associated bacteria in mice subjected to GI candidiasis that was treated with LRE, WLE, or the combination. Although the content of colonic mucosa-associated bacteria as a whole and that of the phylum Firmicutes were unaffected by LRE and WLE administered separately or in combination (Fig. 4A), the abundance of protective bacteria such as *Bifidobacterium* spp. and Faecalibacterium prausnitzii increased after administration of the two extracts combined. In line with this observation, the administration of LRE and WLE in combination tended to increase the content of *Lactobacillus* spp and L. murinus, which is described as a key beneficial bacteria for the health of the intestinal mucosa (28, 29). At the same time, LRE and WLE separately or in combination significantly reduced Bacteroidetes and *Clostridium* spp. loads, which are often increased in dysbiosis. The content of Enterobacteriaceae was unaffected by the extracts (Fig. 4B). Thus, the LRE/WLE combination significantly shifted the composition of gut microbiota toward a protective profile.

### Oral administration of LRE and WLE separately or in combination reduced gut inflammation and improved the oxidative status of colonic tissues of C. albicans-infected mice.

**FIG 5:**
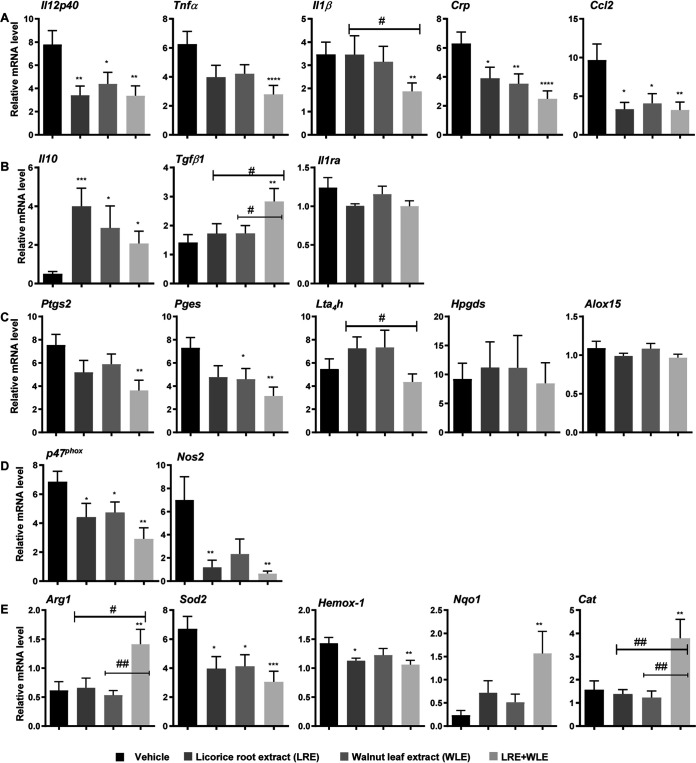
Modulation of colonic inflammatory and oxidative status of C. albicans-infected mice treated with licorice root extract (LRE) and walnut leaf extract (WLE) alone or in combination. LRE and WLE alone or in combination, or vehicle were orally administered to mice (*n* = 10 per group) for 12 days. After this treatment, mice were orally infected with C. albicans and sacrificed 5 days later. Total RNAs isolated from the colon were subjected to the RT-PCR analysis using specific primer sets for (A) proinflammatory markers (*Il12p40* [Interleukin-12p40], *Tnfa* [Tumour Necrosis Factor alpha], *Il1b* [Interleukin-1 beta], *Crp* [C-reactive protein], *Ccl2* [C-C Motif Chemokine Ligand 2]), (B) for anti-inflammatory cytokines (*il10* [Interleukin-10], *Tgfb1* [Transforming Growth Factor Beta 1], *il1ra* [Interleukin-1 receptor antagonist]), (C) for enzymes involved in the production of pro- or anti-inflammatory eicosanoids (*Ptgs2* [cyclooxygenase-2], *Pges* [prostaglandin E synthase], *Lta4h* [LTB4 hydrolase], *Hpgds* [prostaglandin D synthase], *Alox15* [12/15-lipoxygenase]), (D) for pro-oxidant enzymes (*p47^phox^* [a cytosolic subunit of the NADPH oxidase complex], *Nos2* [inducible nitric oxide synthase]), and (E) for enzymes involved in anti-oxidant activities (*Arg1* [arginase-1], *Sod2* [superoxide dismutase], *Hemox-1* [hemoxygenase 1], *Nqo1* [NADPH quinone dehydrogenase 1], *Cat* [catalase-1]). Data are presented as means ± SEM. *, *P* ≤ 0.01; **, *P* ≤ 0.01; ***, *P* ≤ 0.005; ****, *P* ≤ 0.001 compared to vehicle. ^#^, *P* ≤ 0.05; ^##^, *P* ≤ 0.01 compared between treatments.

To investigate the effect of LRE and WLE alone or in combination on colonic inflammation in mice infected with C. albicans, we assessed the expression of proinflammatory and anti-inflammatory markers in colonic tissues. Administration of the plant extracts separately or in combination decreased proinflammatory gene expression (*Il12p40*, *Tnfa*, *Il1b*, *Crp*, *Ccl2*). Interestingly, the combination of the two extracts induced a more robust decline in the expression of proinflammatory genes than either extract administered separately (Fig. 5A). These findings were corroborated by the reciprocal increase in the expression of *IL-10* and *TGF-β*1 anti-inflammatory cytokines in colonic tissues of C. albicans-infected mice that received the plant extracts (Fig. 5B).

Consistent with the decrease in proinflammatory markers induced, the LRE/WLE combination also decreased the mRNA expression of enzymes involved in the synthesis of proinflammatory eicosanoids (*Ptgs2* [cyclooxygenase-2], *Pges* [prostaglandin E synthase] and *LTA4h* [LTB4 hydrolase], a critical enzyme for synthesis of the proinflammatory mediator LTB4) (Fig. 5C). The mRNA expression of enzymes involved in the synthesis of anti-inflammatory eicosanoids (*Hpgds* [prostaglandin D synthase] and *Alox15* [12/15-lipoxygenase]) was not affected by administration of the plant extracts (Fig. 5C).

Regarding the oxidative stress status of the colon, the mRNA expression of *p47^phox^*, a cytosolic subunit of the NADPH oxidase complex, and the expression of inducible nitric oxide synthase (*Nos2*), the activation of which is essential for the release of reactive oxygen species (ROS), were downregulated in response to the plant extracts with a stronger effect when the two extracts were combined (Fig. 5D). In accordance with this reduced oxidative status, the LRE/WLE combination shifted the balance between *Nos2* and arginase-1 toward the expression of arginase-1 (Fig. 5D and E). Moreover, although administration of the LRE/WLE combination slightly decreased *Sod2* (superoxide dismutase) and *Hemox-1* (hemoxygenase 1) concentrations, the combination significantly increased expression of the antioxidant enzymes *Nqo1* (NADPH quinone dehydrogenase 1), *Cat* (catalase-1) and *Arg1* (arginase-1) (Fig. 5E).

Altogether, these data highlight the anti-inflammatory and antioxidant potential of the LRE/WLE combination in the colon during gastrointestinal infection with C. albicans.

## DISCUSSION

Although *Candida* spp. form part of the commensal microbiota in most individuals with a healthy immune system, variations in the local microenvironment, antibiotic treatment, or alterations in the immune system can favor dysbiosis and rapid proliferation of *Candida*, which can then become a pathogen (1, 2). The high incidence of fungal infections caused by *Candida* species and their increasing resistance to antimicrobial treatments, stimulate alternative approaches and new prophylactic therapies.

In the present study, we evaluated the effects of hydroethanolic extracts of licorice root (LRE) and walnut leaf (WLE), administered separately or in combination, on GI colonization by C. albicans, colon inflammation, and gut microbiota composition in mice. We observed that the level of C. albicans in the feces and colonization of the GI tract of infected mice treated with the plant extracts were significantly reduced. Interestingly, the two plant extracts administered together substantially decreased the number of viable C. albicans in the feces as well as the *Candida* burden in the esophagus, cecum, and colon, suggesting that the combination administered orally has synergistic effects and favors the clearance of C. albicans from the GI tract. In previous studies, licorice root extracts and specific compounds have consistently been shown to play a protective role against candidiasis in mice, owing to their ability to modulate the immune system and possibly to their prebiotic effects (11–15, 17, 24–26). Glycyrrhizin administered to mice inoculated with C. albicans at lethal doses decreased the mortality rate by 35%, increased mean survival time from 7 to 11 days, and decreased symptom severity (11). These effects were supported by the results of a study in MAIDS mice, which exhibit a 100 times greater susceptibility to C. albicans infection than wild-type mice, demonstrating the potential of glycyrrhizin to increase their resistance against C. albicans infection (12). In mice immunized with a C. albicans surface mannan extract in emulsion form, 18β-glycyrrhetinic acid (aglycone of glycyrrhizin) exerted a dominant Th1-immunological adjuvant effect (13). *In vitro*, this component also inhibited the growth of C. albicans isolated from patients with recurrent vulvovaginal candidiasis (14). Several additional studies have demonstrated the value of other licorice root compounds in fungal infections (13, 15–17). *In vitro*, the chalcone licochalcone A and the isoflavonoid glabridin showed antifungal activity against C. albicans (15). Licochalcone A had a significant inhibitory effect on biofilm formation, a key virulence factor, while both licochalcone A and glabridin inhibited the yeast-hyphae transition (15). Glabridin was also shown to induce C. albicans apoptosis *via* the caspase-independent route (16). Liquiritigenin, a flavonoid, increased the survival time of mice infected with C. albicans, this licorice root component protecting the mice against disseminated candidiasis by a CD4^+^ Th1 immune response (17).

Thus, the observed antifungal properties of the licorice root extract (LRE) evaluated in the present study could be due to the presence of flavonoids, such as glabridin and liquiritigenin, and to the presence of glycyrrhizic acid (and its derivative glycyrrhetinic acid), identified by UHPLC-MS analysis, and known to increase the resistance of mice to C. albicans infection (12, 13).

As we previously reported, the WLE tested contains several flavonoids, including quercetin, myricetin, kaempferol, and taxifolin derivatives as well as hydroxycinnamic acids (30). In a recent study, an extract of Trachyspermum ammi seeds enriched in rosmarinic acid-3-O-glucopyranoside, as well as kaempferol-(coumaroyl glucosyl)-rhamnoside and quercetin-3-O-galactoside, inhibited *Candida in vitro* (31). The anti-fungal effects of quercetin alone have been demonstrated in other *in vitro* studies (32, 33). It was reported that the regulation of quorum sensing by quercetin, isolated from an edible lichen (Usnea longissima), could sensitize resistant C. albicans to fluconazole and thereby enhance the efficacy of this drug. Quercetin enhanced the destruction of C. albicans NBC099 cells by fluconazole and induced cell death. It was also found to strongly suppress the onset of virulence-enhancing processes such as biofilm formation and hyphal development, as well as phospholipase, proteinase, esterase, and hemolytic activities. The sensitization was dependent on the farnesol response generated by quercetin, farnesol being a quorum-sensing compound produced by C. albicans, that is known to regulate the expression of *Candida* virulence factors. In addition, taxifolin was identified as an inhibitor of the transcriptional factors (Tec1 and Rfg1) inducing the hyphal growth responsible for the invasiveness and virulence of C. albicans (34).

As microbiota composition of the GI tract influences the evolution of *Candida* from a commensal to a pathogenic status and that licorice and walnut are described to have prebiotic effects (23–27, 35), we evaluated the effect of LRE and WLE on colonic microbiota in mice with GI candidiasis. Our data indicate a correlation between LRE and WLE supplementation and a shift within the gut microbiome toward a protective profile. The relevant increase in protective bacteria, such as *Bifidobacterium* spp. and Faecalibacterium prausnitzii, known to have probiotic and anti-inflammatory properties (36–39), following oral administration of the extracts in combination as well as the decrease in pathobionts, such as *Clostridium* spp., support a synergic effect of the two extracts. These findings suggest that regular supplementation may provide prebiotic benefits by modifying the composition and diversity of the gut microbiota in such a way as to counteract *Candida* growth.

In line with its antimicrobial properties inhibiting *Candida* colonization of the GI tract and the reorientation of the colonic mucosal microbiota toward protective bacteria, the combination of the two plant extracts also alleviated colonic inflammation. We demonstrated that the *Candida* burden was greatly diminished by the combination, a finding consistent with the higher reduced colonic inflammation. In line with the anti-inflammatory activity of the combination, several components of licorice, including glycyrrhizic acid and isoliquiritigenin, which were detected in our extract, have been reported to have anti-inflammatory, antioxidant and GI tract protective effects (40–43). Likewise, walnut extract exhibits anti-inflammatory activities through nonchlorogenic and chlorogenic acids known for their antioxidant and anti-inflammatory activities (30, 44–46).

Interestingly, the two plant extracts combined presented a synergistic anti-inflammatory effect related to a greater reduction of proinflammatory cytokines and enzymes involved in the synthesis of proinflammatory eicosanoids. Concomitantly, the two plant extracts combined strongly induced the expression of anti-inflammatory cytokines. Furthermore, this combination showed a stronger antioxidant potential resulting from the downregulation of *p47^phox^*, *Nos2*, and the upregulation of antioxidant enzymes in the colonic tissue of infected mice.

Altogether, our results suggest that the two plant extracts combined effectively control GI candidiasis and the associated gut inflammation through their anti-inflammatory and antioxidant properties, and their ability to modulate the composition of the gut microbiota.

This study highlights the significant prebiotic potential of the LRE/WLE combination and suggests that the health benefits of these plant extracts are due, at least in part, to their ability to modulate the gut microbiota, reduce inflammation, and oxidative stress, and protect against opportunistic infection.

## MATERIALS AND METHODS

### Hydroethanolic extracts of licorice root and walnut leaf.

In this study, we evaluated hydroethanolic extracts of licorice (Glycyrrhiza glabra L.) roots and walnut (Juglans regia L.) leaves provided by PiLeJe Laboratoire. The preparation and phytochemical analysis of the hydroethanolic extract of walnut leaves was previously published (30). Briefly, a hydroethanolic extract of fresh walnut leaves (walnut leaf extract [WLE]; PL-NOY-01; PiLeJe Laboratoire, France) was obtained according to a process similar to that used for the licorice root extract described in detail below. In this previously published study, chromatographic analyses had revealed the presence of various flavonoids (including quercetin, myricetin, kaempferol, and taxifolin derivatives) as well as hydroxycinnamic acids (including neochlorogenic acid).

### Preparation of the hydroethanolic licorice root extract.

Licorice (Glycyrrhiza glabra L.) roots were collected in Spain in November 2017. Fresh licorice roots were extracted by 20% to 70% (vol/vol) ethanolic leaching. The extracts were then mixed and concentrated under reduced pressure (100-pascal absolute pressure) at controlled temperature (35 to 45°C). Glycerol was then added to dilute the resulting extract to a final concentration of 5.2% (wt/wt) (referred to as licorice root extract [LRE]; PL-REG-01; PiLeJe Laboratoire, France).

### HPTLC analysis of the LRE.

Standards were diluted in ethanol 70% at a concentration of 0.4 mg/mL for glycyrrhizic acid and in methanol 0.1 mg/mL for formononetin. The LRE without glycerol (1 mL) was diluted in 3 mL of a mixture of ethanol and water (70/30 vol/vol). The resultant solution was shaken and centrifuged for 5 min at 4400 rpm. The supernatant solution was transferred into individual vials and then submitted for HPTLC analysis. In addition,1.8 g of ground licorice roots was extracted with 20 mL of ethanol and water (70/30 vol/vol). The resultant solution was sonicated for 10 min and centrifuged for 5 min at 4400 rpm. The supernatant solution was transferred into individual vials and then subjected to HPTLC analysis.

HPTLC analysis was performed on 200.0 × 100.0 mm silica gel 60 F 254 HPTLC glass plates (Merck, Germany). Standard solutions and samples were applied to the plate as 6.0 mm wide bands using CAMAG Automatic TLC sampler (ATS 4). The equipment comprised a CAMAG horizontal developing chamber, a TLC plate heater, a CAMAG Derivatizer Device, a CAMAG chromatogram immersion device, a CAMAG visualizer, and VisionCATS software. The general chromatography conditions are presented in Table 2.

**TABLE 2:** General chromatography conditions for HPTLC analysis of the licorice root extract

Parameters	Amino acids	Glycyrrhizic acid	Flavonoids and phenolic acids
Distance from lower edge	5 mm	8 mm	5 mm
Distance from left and right edges	15 mm	20 mm	15 mm
Space between bands	8.4 mm	12 mm	8 mm
No. of tracks	21	6	22
Development distance from lower edge	50 mm	70 mm	50 mm
Mobile phase	Butanol, acetone, acetic acid, water (3.5/3.5/1/2) with 40.9 mg of ninhydrin	Ethyl acetate/acetic acid/formic acid/water (30/2/2/4)	Ethyl acetate/acetic acid/formic acid/water (50/5.5/5.5/13)
Derivatization conditions	100°C for 3 min	Spraying (nozzle: yellow, level:4) with 3 mL of 10% sulfuric acid and heating to 100°C for 10 min	110°C for 10 min and dipping (speed: 5, time: 0) with natural product reagent then polyethylene glycol reagent
Visualization	White light	White light	UV light at 366 nm

### LC/MS analysis of the LRE.

Chromatographic analyses (UHPLC) were performed on an Ultimate 3000 RSLC UHPLC system (Thermo Fisher Scientific Inc., MA, USA) coupled to a binary pump (U3000 HPG-3400RS) and a diode array detector. Compounds were separated on an Uptisphere Strategy C18 column (25 × 4.6 mm; 5 μm; Interchim), which was controlled at 40°C. The mobile phase was a mixture of 0.1% (vol/vol) formic acid in water (phase A) and 0.1% (vol/vol) formic acid in acetonitrile (phase B). The gradient of phase A was 100% (0 min), 80% (10 min), 73% (35 min), 30% (50 min), 0% (55 min). The flow rate was 0.8 mL/min, and the injection volume was 5 μL. The UHPLC system was connected to a Q-Exactive Orbitrap (Thermo Fisher Scientific Inc., MA, USA) mass spectrometer, operated in negative and positive electrospray ionization mode. Source operating conditions: 3 kV spray voltage for negative mode and 3.5 kV spray voltage for positive mode; 320°C heated capillary temperature; 475°C auxiliary gas temperature; sheath, sweep, and auxiliary gas (nitrogen) flow rate 60, 18, and 4 arbitrary units, respectively; and collision cell voltage between 20 and 50 eV. Full scan data were obtained at a resolution of 35,000 whereas MS^2^ data were obtained at a resolution of 17,500. Data were processed using Xcalibur software (Thermo Fisher Scientific Inc., MA, USA).

Compounds present in the LRE were characterized according to their retention times, mass spectral data, and comparison with authentic standards when available or with published data.

### Murine model of gastrointestinal candidiasis.

All mouse experiments were performed according to protocols approved by the institutional ethics committee (CEEA122) and the French Ministry of Higher Education, Research, and Innovation (ESRI) under permit number 5412–2016051917498658;2016 to 2020 and renewed under permit number 23558–2020011016561848;2020 to 2025 in accordance with European legal and institutional guidelines (2010/63/UE) for the care and use of laboratory animals. Female C57BL/6 mice aged 8 weeks were purchased from Janvier Labs (France). LRE and WLE were administered orally, separately (2.5 g/kg) or in combination (1.25 + 1.25 g/kg), once daily for 12 days before C. albicans infection and then 5 days after infection. Control groups received only the vehicle (saline solution diluted with glycerol to the same extent as the extracts). Esophageal and GI candidiasis was established by intraesophageal administration of C. albicans at the rate of 50 × 10^6^ blastospores in sterile saline solution per mouse, as described previously (47, 48). Ten mice were included in each experimental group. Stools were collected daily from day 3 to day 5 after infection to quantify viable C. albicans. After 5 days of infection, the mice were sacrificed and the esophagus, cecum, and colon were aseptically removed to evaluate C. albicans colonization, microbiota composition, and inflammatory status.

### Preparation and quantification of viable C. albicans in stools.

The strain of C. albicans used throughout these experiments (sc-5314) was provided by the American Type Culture Collection (ATCC) and was maintained on Sabouraud dextrose agar (SDA; Bio-Rad, Hercules, CA, USA) plates containing gentamicin and chloramphenicol. Growth from an 18 to 24 h SDA culture of C. albicans was suspended in sterile saline solution (NaCl 0.9%) for mice infection (49, 50).

Stools were collected daily from day 3 to day 5 after infection, weighed, and mechanically homogenized in phosphate buffer saline (PBS). Serial dilutions of homogenates were plated on SDA plates containing gentamicin and chloramphenicol for the quantitative determination of the number of C. albicans. Plates were incubated at 37°C for 24 h and the number of colonies was counted to determine the colonies forming unit (CFU)/g of stool.

### Quantification of C. albicans in the gastrointestinal tract and microbiota analysis using real-time PCR.

The esophagus, cecum, and colon dissected from infected mice were crushed using lysing matrix tubes (MP Biomedicals, Illkirsh, France). Tissue sample homogenates were resuspended in BLB lysis buffer (Roche Diagnostics, Meylan, France) for 20 min at room temperature and DNA was purified using a High Pure PCR Template Preparation kit (Roche). RT-PCR was performed on a Light Cycler 480 system using Light Cycler SYBR Green I Master (Roche). For amplicon detection, the Light Cycler DNA SYBR green I kit was used as described by the manufacturer (Roche Diagnostics, Meylan, France). The primers used are listed in Table 3.

**TABLE 3:** Primers used for gut microbiota analysis (68)

Gene	5′–3′ universal name		5′–3′ sequence
*Candida spp.* (69)	sense		TCGCATCGATGAAGAACGCAGC
	antisense		TCTTTTCCTCCGCTTATTGATATGC
*Clostridium spp.* (28)	sense		CGGTACCTGACTAAGAAGC
	antisense		AGTTTYATTCTTGCGAACG
*Bifidobacterium spp.* (28)	sense		GGGTGGTAATGCCGGATG
	antisense		TAAGCGATGGACTTTCACACC
*Lactobacillus spp.* (28)	sense		AGCAGTAGGGAATCTTCCA
	antisense		CACCGCTACACATGGAG
Total bacteria (29)	sense	Eub338F	ACTCCTACGGGAGGCAGCAG
	antisense	Eub518R	ATTACCGCGGCTGCTGG
Bacteroidetes (29)	sense	Bact934F	GGARCATGTGGTTTAATTCGATGAT
	antisense	Bact1060R	AGCTGACGACAACCATGCAG
Firmicutes (29)	sense	Firm934F	GGAGYATGTGGTTTAATTCGAAGCA
	antisense	Firm1060R	AGCTGACGACAACCATGCAC
Enterobacteriaceae (70)	sense	Uni515F	GTGCCAGCMGCCGCGGTAA
	antisense	Ent826R	GCCTCAAGGGCACAACCTCCAAG
F. prausnitzii (71)	sense	Fprau223F	GATGGCCTCGCGTCCGATTAG
	antisense	Fprau420R	CCGAAGACCTTCTTCCTCC
*L. murinus/animalis* (72)	sense		TCGAACGAAACTTCTTTATCACC
	antisense		ATGACCCAGATCATGTTTGA
Genomic actin (73)	sense		ATGACCCAGATCATGTTTGA
	antisense		TACGACCAGAGGCATACAG

To quantify the number of *Candida*, C. albicans cell suspensions were standardized at 10^6^ cells/mL and serially diluted samples of genomic fungal DNA (range: 100 to 10^6^ cells/mL) were used as external standards in each run. Cycle numbers of the logarithmic linear phase were plotted against the logarithm of the concentration of template DNA to evaluate the number of yeasts present in each tissue sample homogenate and normalized to the amount of genomic β-actin.

Semiquantitative RT-PCR was performed with primers that amplify the genes encoding 16S rRNA from specific bacterial groups on DNA isolated from colonic mucosa to evaluate mucosa-associated bacteria colonization. Relative quantity was calculated and normalized to the amount of genomic β-actin.

### Gene expression analysis by reverse transcription and real-time PCR.

mRNA from colonic tissues were prepared and cDNA was synthesized according to the manufacturer’s recommendations (Total RNA Minipreps super kit, BioBasic; Verso cDNA kit, Thermo Fisher Scientific). RT-PCR was performed on a Light Cycler 480 system with Light Cycler SYBR Green I Master Mix (Roche). Serially diluted samples of pooled cDNA were used as external standards in each run for the quantification. GAPDH was used as the housekeeping gene. The primers (Eurogentec), designed with the software Primer 3, were listed in Table 4.

**TABLE 4:** Primer sequences used in qRT-PCR

Gene	5′–3′Sequence	Sequence
*Alox15*	sense	GTTCAGGAACCACAGGGAGG
	antisense	GTCAGAGATACTGGTCGCCG
*Arg1*	sense	CGTGTACATTGGCTTGCGAG
	antisense	TCGGCCTTTTCTTCCTTCCC
*Cat*	sense	ACATGGTCTGGGACTTCTGG
	antisense	CAAGTTTTTGATGCCCTGGT
*CCL2*	sense	AGGTCCCTGTCATGCTTCTG
	antisense	TCTGGACCCATTCCTTCTTG
*Crp*	sense	CGCAGCTTCAGTGTCTTCTC
	antisense	AGATGTGTGTTGGAGCCTCA
*Gapdh*	sense	ACACATTGGGGGTAGGAACA
	antisense	AACTTTGGCATTGTGGAAGG
*Hemox-1*	sense	CACGCATATACCCGCTACCT
	antisense	CCAGAGTGTTCATTCGAGCA
*Hpgds*	sense	GGACACGCTGGATGACTTCA
	antisense	TCCCAGTAGAAGTCTGCCCA
*Il10*	sense	AGGCGCTGTCATCGATTTCT
	antisense	GCTCCACTGCCTTGCTCTTA
*Il12p40*	sense	AGGTCACACTGGACCAAAGG
	antisense	TGGTTTGATGATGTCCCTGA
*Il1ra*	sense	GGCCTAGGTGTCTTCTGCTC
	antisense	GTAAGGGAGTCACTTGGGGC
*Il1b*	sense	CAACCAACAAGTGATATTCTCGATG
	antisense	GATCCACACTCTCCAGCTGCA
*Lta4h*	sense	GTTGACAGCTGAACCCCAGT
	antisense	CGTGCCCTTAGTTCCACATT
*Nos2*	sense	TCCTGGACATTACGACCCCT
	antisense	ACAAGGCCTCCAATCTCTGC
*Nqo1*	sense	TTCTCTGGCCGATTCAGAGT
	antisense	GGCTGCTTGGAGCAAAATAG
*Pges*	sense	CCTAGGCTTCAGCCTCACAC
	antisense	CAGCCTATTGTTCAGCGACA
*Ptgs2*	sense	AGAAGGAAATGGCTGCAGAA
	antisense	GCTCGGCTTCCAGTATTGAG
*p47phox (Ncf1)*	sense	AGTGATGCGGAGACTTTGCT
	antisense	ACCGGAGTTACAGGCAAATG
*Sod2*	sense	GCCCCCTGAGTTGTTGAATA
	antisense	AGACAGGCAAGGCTCTACCA
*Tgfb1*	sense	AGGTTGGCATTCCACTTCAC
	antisense	AGGGGCCTCTAAGAGCAGTC
*Tnfa*	sense	AGCCCCCAGTCTGTATCCTT
	antisense	CTCCCTTTGCAGAACTCAGG

### Statistical analysis.

GraphPad Prism (GraphPad Software, Inc., La Jolla, CA, USA) was used for graph preparation and statistical evaluation. Differences between groups were assessed using ANOVA, followed by a nonparametric Mann-Whitney test. Differences with *P* ≤ 0.05 were considered significant (*, *P* ≤ 0.05; **, *P* ≤ 0.01; ***, *P* ≤ 0.001; ****, *P* ≤ 0.0001). Data represent mean values ± standard error of the mean (SEM).
